# Dietary pyrroloquinoline quinone hinders aging progression in male mice and D-galactose-induced cells

**DOI:** 10.3389/fragi.2024.1351860

**Published:** 2024-02-29

**Authors:** Nur Syafiqah Mohamad Ishak, Midori Kikuchi, Kazuto Ikemoto

**Affiliations:** Niigata Research Laboratory, Mitsubishi Gas Chemical Company, Inc., Niigata, Japan

**Keywords:** PQQ, inflammation, aging, antioxidant, sarcopenia, muscle atrophy

## Abstract

**Background:** Understanding and promoting healthy aging has become a necessity in the modern world, where life expectancy is rising. The prospective benefits of the antioxidant pyrroloquinoline quinone (PQQ) in healthy aging are promising. However, its role in aging remains unclear. Thus, this study aimed to investigate the effect of PQQ on preventing the progression of aging and to explore its underlying molecular mechanisms.

**Methods:** Naturally aged C57BL/6J male mice were fed a normal diet with or without PQQ (20 mg/kg/day) for 10 weeks. Body composition was measured by bioimpedance at weeks 0 and 8. The integument conditions were evaluated at weeks 0, 4, and 8. Muscle strength and function were examined at week 8. At the ninth week, computed tomography images of the mice were captured, and blood and tissue samples were collected. The levels of inflammatory cytokines in the gastrocnemius muscle were measured, and the muscle fiber cross-sectional area in the soleus muscle was examined. Additionally, a D-galactose (D-gal)-induced cell aging model was used to study the effects of PQQ intervention on cell proliferation, senescence, differentiation, ROS levels, and mitochondrial function in myoblasts (C2C12). Cell proliferation and monolayer permeability of D-gal-induced intestinal epithelial cells (IEC6) were also examined.

**Results:** Aged mice suffered from malnutrition; however, PQQ supplementation ameliorated this effect, possibly by improving metabolic dysfunction and small intestinal performance. PQQ prevented rapid loss of body fat and body fluid accumulation, attenuated muscle atrophy and weakening, reduced chronic inflammation in skeletal muscles, and improved skin and coating conditions in aged mice. Furthermore, PQQ intervention in D-gal-treated C2C12 cells improved mitochondrial function, reduced cellular reactive oxygen species (ROS) levels and senescence, and enhanced cell differentiation, consequently preventing age-related muscle atrophy. In addition, PQQ increased cell proliferation in D-gal-treated IEC6 cells and consequently improved intestinal barrier function.

**Conclusion:** PQQ could hinder the aging process and particularly attenuate muscle atrophy, and muscle weakness by improving mitochondrial function, leading to reduced age-related oxidative stress and inflammation in muscles. PQQ may also ameliorate malnutrition caused by intestinal barrier dysfunction by enhancing IEC proliferation. This study provides evidence for the role of PQQ in aging and suggests that PQQ may be a potential nutritional supplementation that can be included in healthy aging strategies.

## 1 Introduction

With a significant increase in the number of older adults in the global population, understanding and promoting healthy aging has become crucial. The World Health Organization (WHO) defines healthy aging as the process of retaining functional capacity in older age that enables overall wellbeing (WHO, 2015). Thus, the health of older adults should be a priority, indicating the need for urgent action. Aging is defined as the progressive loss of tissue and organ function. It is linked to changes in body composition, particularly fat mass, and fat-free mass, which may have negative health consequences (Pappas and Nagy, 2019). Several age-related conditions, including sarcopenia and frailty, are caused by common components of the aging process, which are chronic inflammation, also known as “inflammaging,” and oxidative stress (Liguori et al., 2018; Antuña et al., 2022). Studies have shown that excess oxidative stress and DNA damage activate inflammasomes, stimulating the nuclear factor-κB (NF-κβ) and interleukin-1β (IL-1β)-mediated inflammatory pathways (Rea et al., 2018). Food-derived antioxidants such as resveratrol and selenium have been shown to reduce inflammaging by resolving the redox imbalance associated with aging (Zhou et al., 2021; Bjørklund et al., 2022). Thus, it is commonly believed that antioxidants are key players in alleviating age-related conditions.

Pyrroloquinoline quinone (PQQ), an antioxidant found in various foods and in human breast milk (Kumazawa et al., 1995; Mitchell et al., 1999), has recently gained attention in public health. PQQ was first discovered as a cofactor of methanol dehydrogenase (Hauge, 1964). It is an aromatic tricyclic orthoquinone with redox cycling that has 7.4 times the aroxyl radical-scavenging capacity of vitamin C in its reduced form, proving that it is the most potent water-soluble antioxidant (Akagawa et al., 2016). PQQ has shown potential health benefits owing to its growth-promoting, anti-diabetic, anti-obesity, and neuroprotective functions (Jonscher et al., 2021; Mohamad Ishak and Ikemoto, 2023). PQQ is currently manufactured as a red powder through fermentation and can be used as a supplement in the form of disodium salt (BioPQQ^®^, C_14_H_10_N_2_Na_2_O_11_, formula weight: 428.22 g/mol) since it has been approved as a food ingredient in the United States in 2008 and in Japan in 2014. After passing the European Food Safety assessment, the European Commission also approved this product as a novel food ingredient in 2018 (Turck et al., 2017).

Based on the following properties, the potential benefits of PQQ in healthy aging appear promising. First, PQQ is known to promote mitochondrial function (Saihara et al., 2017). Mitochondria are the powerhouses of cells responsible for energy production. With age, mitochondrial function tends to decline, contributing to various health issues (Sun et al., 2016). PQQ supports mitochondrial biogenesis, which could potentially help improve overall cellular energy production and function during aging. Second, PQQ exhibits potent antioxidant properties (Miyauchi et al., 1999; He et al., 2003), indicating that it can help neutralize harmful free radicals in the body. Excessive oxidative stress and damage caused by free radicals are associated with aging and age-related diseases (Liguori et al., 2018). PQQ may contribute to healthy aging by reducing oxidative stress. Third, PQQ exhibits anti-inflammatory properties, which can mitigate the effects of chronic inflammation (Liu et al., 2016; Ma et al., 2019). Chronic inflammation also contributes to aging and age-related diseases (Rea et al., 2018). However, evidence supporting the beneficial role of PQQ in aging is still limited.

In this study, we examined the role and mechanism of action of dietary PQQ used as a nutritional intervention in preventing the progression of natural aging. We investigated the effects of PQQ on aged mice by examining changes in their body composition, integument conditions, muscle atrophy, and function. Furthermore, we explored the molecular mechanism underlying aging progression using a cell culture induced by D-galactose (D-gal) as an aging model system. This study provides insights into the mechanism whereby PQQ inhibits oxidative stress via the improvement of mitochondrial function, subsequently preventing inflammaging. This study also provides evidence that PQQ is an effective anti-aging component to include in healthy aging measures.

## 2 Materials and methods

### 2.1 Ethics statement

All experimental procedures were approved by and conducted in accordance with the Animal Care and Experimentation Facility Committee of the Analysis Center, Mitsubishi Gas Chemical Company (Approval No. NA23-01). This study was designed based on the ARRIVE guidelines (Percie du Sert et al., 2020) and adhered to all legal requirements of the Japanese regulations of laboratory animal care and experiments with 3Rs.

### 2.2 Animal experiments

**FIGURE 1:**
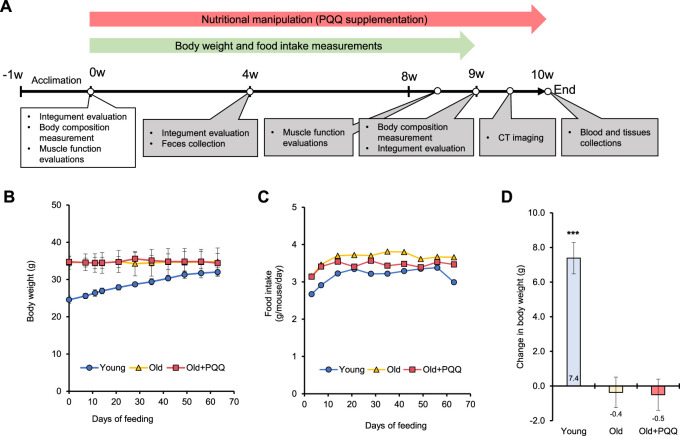
**(A)** Schematic illustrating the timeline of the animal experiments. After 1 week of acclimation, the old + PQQ group was provided with PQQ supplementation for 10 weeks, and body weight and food intake measurements were performed every week until week 9 of the experimental period. The white box indicates the measurements performed during the starting point, whereas gray boxes indicate the measurements performed after nutritionary supplementation started. **(B)** Body weight and **(C)** food intake per mouse of each group were measured each week. The average of food intake and PQQ intake per mouse per day for each group are presented in Table 1. **(D)** Change in body weight after 2 months of feeding. The data indicate mean ± SEM (n = 6 per group). Asterisks denote a statistically significant difference (Paired *t*-test; ***p < 0.001) when comparing the data between the starting point and after 2 months of feeding for each group. Bars without asterisks denote statistical non-significance.

Young (8 weeks old) and old (83 weeks old) C57BL/6J male mice were purchased from the Jackson Laboratory Japan (Yokohama, Japan) and housed in an animal facility under constant temperature (20°C–26°C), humidity (40%–70%), and light/dark cycle (12/12 h). The mice were kept in cages (width × depth × height, 213 × 324 × 131 mm; CLEA Japan, Tokyo, Japan) with each cage containing either three young mice or two old mice. Mice were provided *ad libitum* AIN-93M diet pellets (Oriental Yeast, Tokyo, Japan) and water*.* After a week of acclimation, the mice were assigned to three groups (n = 6 in each group): young, old, and old + PQQ. To minimize the effects of confounding factors and create a balanced comparison, old mice were allocated to each group such that they had similar mean values for initial body weight and body fat percentage. For the old + PQQ group, the diet was changed after the acclimation period to AIN-93M mixed with 0.02% (*w/w*) PQQ (BioPQQ, Mitsubishi Gas Chemical, Tokyo, Japan) pellets prepared by Oriental Yeast Co. Inc. We selected this dose of PQQ because it has shown to be effective in our previous study on obesity (Mohamad Ishak et al., 2021). The average PQQ intake by the old + PQQ group was 19.9 mg/kg body weight per day. The body weight of each mouse and the food intake per cage were measured every time the food pellets were renewed, which was twice per week, and the total experimental period was 9 weeks. A schematic of the experimental timeline is shown in Figure 1A.

### 2.3 Mice integument evaluation

Mouse frailty of skin conditions was assessed based on the clinical symptoms of alopecia, dermatitis, and coating state (Whitehead et al., 2014). Mouse integument conditions were evaluated at weeks 0, 4, and 9 under anesthesia and scored using the parameters shown in Supplementary Table S1.

### 2.4 Measurement of body composition

The body composition of each mouse was examined under anesthesia with 3% isoflurane using an ImpediVet bioimpedance spectroscopy device (ImpediMed Limited, Brisbane, Australia), according to manufacturer’s instructions (Smith et al., 2009). The measurements were performed at the start (0 weeks) and end point (9 weeks) of the experiment. The body weight and body length of each animal were measured before bioimpedance spectroscopy measurements. Measurements were conducted continuously in triplicates with 10-s intervals for each mouse, and the average fat mass and total body water values were used for data analysis.

### 2.5 Computed tomography (CT) imaging of body fat

After anesthetizing the mice with 3% isoflurane, CT imaging was performed using NAOMi-CT-3D (RF Co., Ltd., Nagano, Japan) at a voltage of 70 kV and current of 5 mA and with a copper plate X-ray filter and normal mode setting. CT images were visualized using the CT Viewer software (RF Co., Ltd.).

### 2.6 Muscle function evaluation

Hanging and pole tests were performed to evaluate muscle function in mice. The tests were conducted at the start (week 0) and end points (middle of week 8) of the experiment.

#### 2.6.1 Hanging test

A four-limb hanging test was performed to measure muscle strength in mice (Bonetto et al., 2015) using a single chamber of wire hanging test (WH-3002; width × depth × height, 200 × 220 × 330 mm; O’hara and Co. Ltd., Tokyo, Japan). Each mouse was placed at the center of the grid, and then, the grid was turned upside down. The inverted hanging time was measured as the time the mouse could stay hanging before falling within a maximum time of 20 min. Measurements were taken in triplicate for each mouse with an interval of more than 30 min between tests.

#### 2.6.2 Pole test

The pole test was performed to measure motor dysfunction caused by aging in mice (Kurosaki et al., 2003; Balkaya and Endres, 2010). The test assessed the ability of a mouse to grasp and maneuver on a pole until it descended to the floor. The mouse was placed head-upward on top of a 50 cm vertical pole with a diameter of 10 mm. The time until the animal turned face downward completely (T_turn_) and the time until it reached the floor (Total_total_) were measured in triplicate for each mouse with more than 30 min intervals between tests. The trial was repeated when the animal paused during the descent. When the animal slid down from the pole, the ratio of the sliding distance of the pole was scored as follows: 0, no sliding; 1, 1/3 sliding; 2, 1/2 sliding; and 3, 2/3 sliding. Animal training was performed a day before the testing day.

### 2.7 Blood and tissue collection

Blood and tissue samples were collected from each mouse at the end of the experiment. Blood was collected from the inferior vena cava using a 25G needle syringe (Terumo, Tokyo, Japan) under 3% isoflurane anesthesia. Each blood sample was dispensed into a collection tube containing a Capiject II serum separator and a coagulation promoter (Terumo) and mixed by shaking the tube 10 times. The mixtures were allowed to stand at room temperature (22°C–26°C) for a maximum of 2 h before centrifugation at 2,900 × *g* at 20°C for 10 min. The supernatants were transferred into a 1.0 mL tube and stored at −80°C until serum analysis was conducted. After blood removal, the gastrocnemius muscle and soleus muscle of each mouse were collected for gene expression analysis and histopathological examination, respectively. The tissue samples for gene expression analysis were submerged in a DNA/RNA shield solution (Zymo Research, CA, United States), whereas those for histopathological examination were submerged in 4% paraformaldehyde (FujiFilm Wako, Japan). All tissue samples were stored at 4°C until the analyses were performed.

### 2.8 Serum analyses

Serum analyses were performed by Oriental Yeast Co. Inc. Serum albumin (ALB), total cholesterol (T-CHO), triglyceride (TG), creatinine (CRE), creatine kinase (CK), aspartate aminotransferase (AST), alanine aminotransferase (ALT), sodium (Na), calcium (Ca), and magnesium (Mg) levels were measured for each sample. The mean ± standard error of the mean (SEM) was calculated for each mouse group.

### 2.9 Gene expression analysis

The tissues were cut into smaller pieces, and less than 50 mg of tissue was transferred into 2.0 mL sample tubes (TOMY SEIKO, Tokyo, Japan) containing an appropriate amount of silicon beads (TOMY SEIKO). The homogenization was performed using the Micro Smash machine MS-100 (TOMY SEIKO) in the presence of DNA/RNA shield solution (Zymo Research, CA, United States). After homogenization, the tissue samples were incubated with proteinase K at room temperature for 2–3 h. RNA was extracted using Quick-RNA Miniprep (Zymo Research) following the manufacturer’s protocol.

The RNA samples were diluted with DNase/RNase Free water to an appropriate concentration, and 40–100 ng of RNA was used as a template in a RT-qPCR experiment using the One-Step TB Green PrimeScript PLUS RT-PCR kit (Takara Bio, Shiga, Japan). The reactions were performed using the Thermal Cycler Dice Real Time System (Takara Bio) with thermal setting as follows: pattern 1 (reverse transcription) 42°C for 5 min and 95°C for 10 s; pattern 2 (PCR amplifications) cycles of 40°C and 95°C for 5 s and 60°C for 30 s; and pattern 3 (dissociation): 95°C for 15 s, 60°C for 30 s, and 95°C for 15 s. The relation quantification of gene expression was calculated using the 2^−ΔΔCT^ method (Livak and Schmittgen, 2001) with *B-actin* as a reference gene and RNA from the young mice as a reference sample. The primer sets used in this study are listed in Supplementary Table S2.

### 2.10 Muscle fiber cross-sectional area (CSA) examination

The soleus muscle was used to evaluate the number and size of fast and slow myosin muscle fibers. Paraffin sections were prepared by fixing soleus muscle of each mouse in cold 4% paraformaldehyde. The tissue sections were incubated with Anti-Fast Myosin Skeletal Heavy chain antibody (Abcam, MA, United States, Cat # ab91506, RRID: AB_10714690) and Anti-Slow Myosin Skeletal Heavy chain antibody (Abcam, Cat # ab234431, RRID: AB_3076242) to stain fast and slow muscle fibers, respectively. Detection was performed using a horseradish peroxidase-conjugated compact polymer system, with DAB (Abcam) as the chromogen. The preparation of histopathological specimens and immunohistochemical staining were performed by KAC Co., Ltd. Images of the immuno-stained tissue specimens were captured using an optical microscope (Nikon Eclipse TC2000-S; Nikon, Japan) equipped with a digital microscope camera (Nikon Ds-Ri2). Two images of the whole tissue section captured at a magnification of ×4 for each of the six animals per group were analyzed using the ImageJ (RRID:SCR_003070) software to measure the number and CSA of positive immuno-stained myofibers in the tissue sections.

### 2.11 Cell culture and treatments

#### 2.11.1 Myoblast C2C12 cells

The mouse myoblast cell line C2C12 was provided by RIKEN BRC through the National BioResource Project of the Ministry for Education, Culture Sports, Science and Technology, Japan. C2C12 cells were grown in low-glucose Dulbecco’s Modified Eagle Medium (DMEM) supplemented with 10% fetal bovine serum (FBS) (Thermo Fisher Scientific, MA, United States), 100 U/mL penicillin and 100 μg/mL streptomycin (Pen-Strep, FujiFilm Wako, Kanagawa, Japan). The cells were maintained at 37°C in 5% CO_2_. The cells were treated with 20 g/L D-gal for 24 h after seeding to mimic aging conditions, the lowest dose that has been shown to induce senescence and cell apoptosis in the C2C12 cell line (Chen et al., 2020). The effect of PQQ was investigated by adding PQQ (25, 50, and/or 100 nM) simultaneously during the D-gal treatment.

To induce cell differentiation into myotubes, the culture media of C2C12 myoblasts at 80%–90% confluency were changed to DMEM high glucose supplemented with 2% bovine serum (Thermo Fisher Scientific) and 1% Pen-Strep. Fully differentiated myotubes were obtained around day 9 of myoblast cell culture. The cells were maintained at 37°C in 5% CO_2_, and the medium was changed three times per week.

#### 2.11.2 Small intestine epithelial IEC6 cells

The rat small intestine epithelial IEC6 cells were provided by the RIKEN BRC through the National BioResource Project of the MEXT, Japan. The IEC6 cell line was grown in low-glucose DMEM supplemented with 5% FBS (Thermo Fisher Scientific), 4 μg/mL insulin (Sigma Adrich, MA, United States), and 1% penicillin and streptomycin (Pen-Strep, FujiFilm Wako). The cells were maintained at 37°C under 5% CO_2_. PQQ was added (0.1–50 µM) simultaneously during D-gal (20 g/L) treatment.

### 2.12 Cell proliferation, cellular senescence, and cell differentiation tests

Cell proliferation was measured 24 or 48 h after treatment with the Cell Counting Kit-8 (CCK-8; Dojindo, Tokyo, Japan). After washing the treated cells with media, the cells were incubated in culture media supplemented with 10% CCK-8 solution at 37°C for 1 h. The absorbance was measured at 450 nm using a microplate reader (Multimode Plate Reader ARVO X3; PerkinElmer, CT, United States).

Cellular senescence was evaluated by measuring senescence-associated β-galactosidase (SA-β-gal) activity using Cellular Senescence Plate Assay Kit–SPiDER-βGal (Dojindo) 48 h after treatment. The measurements were normalized by nuclei Hoechst 33,342 staining using the Cell Count Normalization Kit (Dojindo). The fluorescence intensity was measured using a fluorescence microplate reader equipped with a 485/535 nm filter (green) for SA-β-gal and 355/460 nm filter (blue) for cell nuclei count.

Cell differentiation was evaluated by myosin heavy chain (MHC) immunostaining. Differentiated myoblasts or myotubes were fixed in 4% paraformaldehyde in PBS for 10 min and permeabilized with 0.5% TritonX-100 in PBS for 5 min. After washing with PBS, the myotubes were treated with 1% BSA in PBS for 15 min. The samples were then incubated with primary mouse monoclonal anti-MHC antibody (R&D Systems, MN, United States of America, Cat# MAB4470, RRID: AB_1293549) for 2 h, followed by a secondary antibody reaction with Alexa Fluor 647 goat-anti mouse IgG (ab150115). ProLong Gold Antifade Mountant with DAPI (Invitrogen) was used to stain the cell nuclei. Images of fluorescence-stained cells were captured using a fluorescence microscope (Nikon, Tokyo, Japan, Nikon Eclipse TC2000-S) equipped with a digital microscope camera (Nikon, Nikon Ds-Ri2). The images were analyzed using the ImageJ software with a macro package called ViaFuse (Hinkle et al., 2021) to quantify skeletal muscle cell viability and differentiation. The ratio of the number of nuclei in myotubes to the total number of nuclei (fusion index, FI) was calculated as a measurement of cell differentiation. FI indicates the percentage of myoblasts that differentiate into myotubular cells by counting the presence of two or more nuclei in a single myotube.

### 2.13 Reactive oxygen species (ROS) levels and mitochondria measurements

Total cellular ROS level in myoblast cells was measured with ROS-ID Total ROS detection kit (Enzo Life Science, Inc., NY, United States) 24 h after treatment. The cells were stained with Oxidative Stress Detection reagent and incubated at 37°C for 1 h. The stained cells were analyzed using a fluorescence microplate reader equipped with a 485/535 nm filter and observed under a fluorescence microscope.

Mitochondria were monitored by staining with MitoBright Green LT dye (Dojindo) and analyzed based on its fluorescence intensity. After staining the myoblast cells for 30 min, they were washed with PBS, followed by adding DMEM without phenol red. The cells were then observed over time under a fluorescence microscope. Images of fluorescence-stained cells at 24 and 72 h after treatment were captured under a GFP filter using a fluorescence microscope (Nikon Eclipse TC2000-S) equipped with a digital microscope camera (Nikon Ds-Ri2). The images were analyzed using the ImageJ software to measure mitochondrial density.

### 2.14 Monolayer permeability assay

IEC6 cells (2×10^5^ cell/well) were grown until the cells formed a monolayer in Transwell membranes (Cell Culture Insert, Corning, NY, United States of America), which were placed in a 12-well plate. The cells were then exposed to 20 g/L D-gal or D-gal with the addition of 50 µM PQQ for 48 h. After the treatment, trans-endothelial electrical resistance was measured using the Millicell-ERS (Electrical Resistance System, Merck, Germany) according to the manufacturer’s instructions. Next, 1 g/L FITC-dextran (4 kDa) was applied to the Transwell membranes and incubated for 1 h. Aliquots of 100 µL of sample from each well were transferred into a 96-wells plate. The fluorescence intensity was measured using a microplate reader with a 485/535 nm filter.

### 2.15 Statistical analysis

Statistical analyses were performed using the SigmaPlot (RRID: SCR_003210) 15.0 software. One-way ANOVA was used for all pairwise multiple comparison procedures with the Holm–Sidak method or Paired *t*-test method for appropriate experiments.

## 3 Results

### 3.1 Mouse body weight and food intake

We examined the changes in body weight (Figure 1B) and food consumption of mice (Figure 1C) during the study period. The young group showed a normal significant increase (*p* < 0.001) in body weight, whereas the old and old + PQQ groups showed no significant changes in body weight after 2 months (Figure 1D). However, there were significant differences in average food intake between the young, old, and old + PQQ groups as shown in Table 1. Food intake in the young group increased during the first 3 weeks and then remained the same throughout the next 5 weeks before decreasing in the final week. Additionally, the old and old + PQQ groups had a higher food intake level than the young group did at the beginning of the test. Food intake increased for the first 2 weeks and remained unchanged thereafter for the old group; however, for the old + PQQ group, the food intake amount only increased in the first week before it became constant throughout the experiment.

**TABLE 1:** Food intake and body changes of each group during the experiment. 0M (0 months) indicates data at the starting point of the experiment, while 2M (2 months from the starting point) indicates data at the end point of the experiment. Data indicate the mean ± SEM (n = 6 per group). Values with different superscript letters in a row are significantly different among groups (one-way ANOVA, all pairwise multiple comparisons with the Holm–Sidak method, *p* < 0.05), whereas the values without superscript letters are non-significant to each other.

	Young	Old	Old + PQQ	*p* Values		
				Young vs old	Young vs old + PQQ	Old vs old + PQQ
Food intake per mouse per day (g)	3.2 ± 0.07^a^	3.6 ± 0.06^b^	3.4 ± 0.03^c^	<0.001	0.002	0.017
PQQ intake per kg body weight per day (mg)	0^a^	0^a^	19.9 ± 0.4^b^	<0.001	<0.001	1.000
Body weight (g), 0M	24.6 ± 0.4^a^	34.6 ± 0.7^b^	34.8 ± 0.5^b^	<0.001	0.806	<0.001
Body weight (g), 2M	31.9 ± 0.6	34.2 ± 1.6	34.3 ± 1.1	0.309	0.195	0.976
Fat mass (g), 0M	11.0 ± 0.5	12.4 ± 1.2	12.2 ± 0.8	0.255	0.408	0.902
Fat mass (g), 2M	12.0 ± 0.8^a^	8.6 ± 1.6^b^	10.2 ± 1.1 ^ab^	0.021	0.132	0.213
Body fat ratio (%), 0M	44.9 ± 1.8^a^	35.7 ± 3.0^b^	35.1 ± 2.4^b^	0.034	0.034	0.853
Body fat ratio (%), 2M	37.5 ± 1.9^a^	24.6 ± 3.5^b^	29.7 ± 2.7 ^ab^	0.015	0.129	0.214
Total body water (%), 2M	45.7 ± 3.4^a^	55.2 ± 6.3^b^	51.5 ± 4.9 ^ab^	0.015	0.127	0.218

### 3.2 Frailty in mouse integument conditions

**FIGURE 2:**
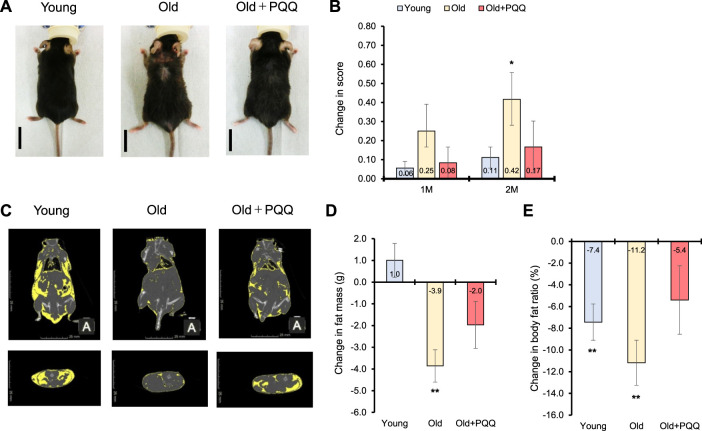
Effects of PQQ on integument conditions and total body fat of aged mice. **(A)** The appearance of representative mice from each dietary group after 2 months of feeding. **(B)** Change in score of the integument conditions, after 1 month (1M), and 2 months (2M) of the experiment. **(C)** The representative CT scan images acquired at week 9 of the study, in which the yellow regions show the distribution of body fat. **(D)** Change in fat mass and **(E)** body fat ratio after 2 months of feeding. Male C57BL/6J were divided into young (8 weeks old), old (83 weeks old), and old + PQQ (83 weeks old) groups, and only old + PQQ mice received dietary supplementation of PQQ. The data indicate mean ± SEM (n = 6 per group). Asterisks denote a statistically significant difference (Paired *t*-test; *p < 0.05) when comparing data between the starting point and after 2 months of feeding for each group. Bars without asterisks denote statistical non-significance.

To assess the frailty of the integument conditions, alopecia, dermatitis, and coating conditions were observed at the beginning of the experiment (0 months) and 1 and 2 months later. The images of each mouse at each time point are shown in Supplementary Figure S1. The integument conditions were scored and averaged (Table 2), with scores closer to 1 indicating a more severe condition. The results showed that aged mice exhibited higher scores at the beginning of the study compared with the young group, and their scores worsened over time. However, PQQ reduced the deterioration of the outer skin condition in aged mice (Figure 2A), as the average score in the old + PQQ group was lower than that in the old group (Table 2). After 2 months, only the old group showed a statistically significant change in score (*p* = 0.03) compared to that at the starting point; this was not seen in the young (*p* = 0.1) and old + PQQ (*p* = 0.3) groups (Figure 2B). Additionally, three out of six mice in the old group had severe condition (score ≥0.7) while others had mild condition (score ≥0.3 and <0.7). In the old + PQQ group, two of six mice had severe condition, one had mild condition, and the others were normal (score <0.3). This result indicates that PQQ treatment slowed down the progress of frailty in mouse integument conditions.

**TABLE 2:** Evaluation of integument conditions. 0M indicates data at the starting point of the experiment (0 months), whereas 1M and 2M indicate data at 1 month and 2 months from the starting point of the experiment, respectively. Data indicate the mean ± SEM (n = 6 per group). Values with different superscript letters in a row are significantly different among groups (one-way ANOVA, all pairwise multiple comparisons with the Holm–Sidak method test, *p* < 0.05).

(M)	Young	Old	Old + PQQ	*p* Values		
				Young vs old	Young vs old + PQQ	Old vs old + PQQ
Score, 0	0 ± 0^a^	0.25 ± 0.0^b^	0.28 ± 0.1^b^	0.018	0.018	1.00
Score, 1	0.06 ± 0.04^a^	0.50 ± 0.13^b^	0.36 ± 0.15 ^ab^	0.044	0.149	0.403
Score, 2	0.11 ± 0.06^a^	0.67 ± 0.13^b^	0.44 ± 0.16 ^ab^	0.020	0.252	0.228

### 3.3 Changes in body composition

At the beginning of the experiment, the average body fat mass of each group was almost the same. The average body fat ratio (percentage of body fat per body weight) was significantly higher for the young group than that for the other groups; however, it was not significantly different between the old and old + PQQ groups (Table 1). The body fat distributions were observed at week 9 of the study using a CT scan (Figure 2C). After 2 months, the old group exhibited a significant reduction in body fat mass (*p* = 0.004). The young group exhibited a slight increase (*p* = 0.3) in body fat mass, whereas the old + PQQ group showed a slight reduction (*p* = 0.1) (Figure 2D). Additionally, all groups showed reductions in body fat ratio (Figure 2E), of which those of the young (*p* = 0.007) and old groups (*p* = 0.003) were significant compared to that at the beginning of the experiment. The amount of water in the body was 46%, 55%, and 52% in the young, old, and old-PQQ groups, respectively (Table 1). The results demonstrated that aged mice experienced rapid body fat reduction and body fluid accumulation due to progress of aging. However, supplementation of PQQ in aged mice substantially suppressed body fat reduction and body fluid accumulation.

### 3.4 Progression of muscle atrophy and weakness

**FIGURE 3:**
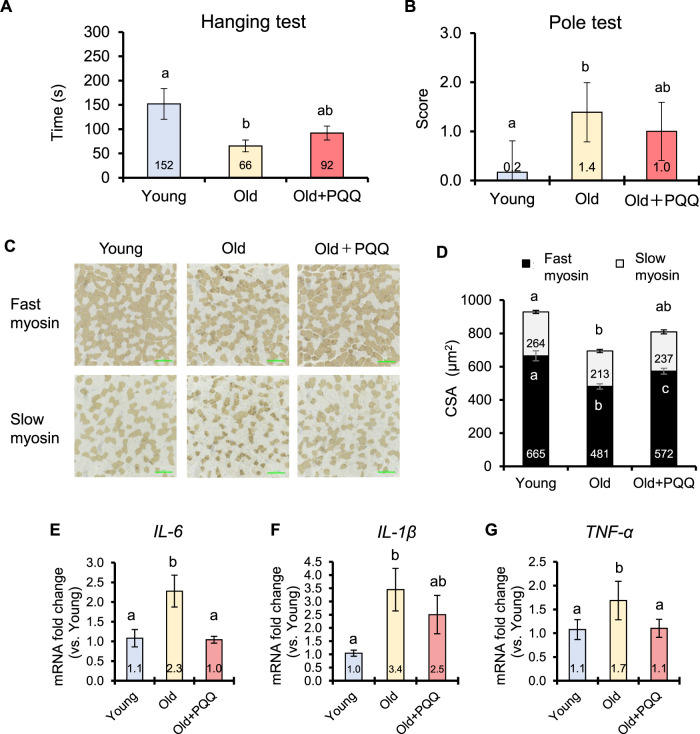
Effects of PQQ on muscle function, muscle atrophy, and inflammaging of aged mice. **(A)** The latency time of each group during the wire hanging test and **(B)** the score of slipping distance during the pole test performed 2 months after the experiment. **(C)** The representative immunohistochemical staining images of fast and slow myosin heavy chains in soleus muscle, in which brown-stained regions show the positive fibers. **(D)** Cross-sectional area (CSA) of fast and slow muscle fibers (μm^2^). Analyses of **(E)** *IL-6* **(F)** *IL-1β*, and **(G)** *TNF-α* cytokines expressions in mouse gastrocnemius muscle. The data indicates mean ± SEM (n = 6 per group). Bars with different letters demonstrate significant differences among groups (one-way ANOVA, all pairwise multiple comparisons with the Holm–Sidak method, *p* < 0.05).

The wire hanging test was performed at the start and end points of the experiment to evaluate muscle strength in mice. At the starting point, the young group had significantly higher latency times than the other groups, whereas latency time was not significantly different between the old and old + PQQ groups (Supplementary Figure S2A). After 2 months of diet consumption, the young group was significantly different from the old group, which exhibited a shorter latency time; however, the old + PQQ group was not significantly different from either the young or old group (Figure 3A). These results demonstrated that aged mice had lower muscle strength than younger mice, and PQQ could attenuate the reduction in muscle strength in aging mice.

The pole test is widely used to evaluate motor deficits associated with the basal ganglia in mice. At the starting point, the T_turn_ was not significantly different among the groups (Supplementary Figure S2B), but the T_total_ of the young group was significantly shorter than that of the old and old + PQQ groups, both of which were not significantly different from each other (Supplementary Figure S2C). After 2 months, most aged mice were unable to grasp the pole and descend properly and partially slipped owing to motor dysfunction; therefore, no significant difference was found in T_turn_ and T_total_ among the groups (Supplementary Figures S2D, E). Thus, we measured the ratio of the sliding distance in the pole. The old + PQQ group showed shorter pole distances than the old group (Figure 3B), suggesting that PQQ prevented the progression of motor dysfunction in aged mice.

### 3.5 Alteration in muscle fibers and chronic inflammation

Muscle atrophy can be defined in relation to muscle fibers by referring to decreases in the size and changes in the structure of muscle fibers. In this study, we evaluated muscle atrophy by measuring the CSA (the size of muscle fibers) and that per number of muscle fibers (the structure of muscle fibers). Skeletal muscle fibers obtained from the soleus muscle were visualized using immunohistochemical staining (Figure 3C). Comparing the young and old groups, we found that the number and CSA of both fast and slow myosin fibers atrophied with age (Table 3; Figure 3D). The number of fast and slow muscle fibers did not change in PQQ-fed aged mice, but the CSA was significantly greater in the fast and total muscle fibers in the old + PQQ group than in the old group. The CSA per muscle fiber in the old + PQQ group was similar to that in the young group. Age-related atrophy of muscle fibers was observed in the soleus muscle; however, PQQ intake significantly reduced fast myosin atrophy. Moreover, although not significant, the old + PQQ showed a trend toward reduced slow myosin atrophy, suggesting that PQQ intake may contribute to improved muscle function and strength.

**TABLE 3:** Number and cross-sectional area (CSA) of myosin fibers. Data indicate the mean ± SEM (n = 6 per group). Values with different superscript letters in a row are significantly different among groups (one-way ANOVA, all pairwise multiple comparisons with the Holm–Sidak method test, *p* < 0.05), whereas the values without superscript letters are non-significant to each other.

	Young	Old	Old + PQQ	*p* Values		
				Young vs old	Young vs old + PQQ	Old vs old + PQQ
Number of fast myosin fibers	632 ± 16^a^	538 ± 27^b^	547 ± 23^b^	0.028	0.033	0.78
CSA of fast myosin fibers (µm^2^)	664.9 ± 30.3^a^	480.7 ± 15.9^b^	572.3 ± 17.5^c^	<0.001	0.020	0.020
CSA per muscle fiber (fast myosin)	1.05 ± 0.04	0.91 ± 0.07	1.05 ± 0.05	0.072	0.965	0.191
Number of slow myosin fibers	293 ± 21	276 ± 8	273 ± 10	0.504	0.363	0.843
CSA of slow myosin fibers (µm^2^)	264.0 ± 9.2^a^	213.2 ± 9.5^b^	237.2 ± 12.2 ^ab^	0.011	0.169	0.169
CSA per muscle fiber (slow myosin)	0.92 ± 0.05	0.78 ± 0.04	0.87 ± 0.04	0.127	0.474	0.166
Number of total fibers	925 ± 23^a^	814 ± 30^b^	820 ± 31^b^	0.040	0.04	0.879
CSA of total fibers (µm^2^)	928.9 ± 29.4^a^	693.9 ± 14.1^b^	809.5 ± 23.4^c^	<0.001	0.005	0.005
CSA per muscle fiber (total fiber)	1.01 ± 0.04^a^	0.86 ± 0.05^b^	0.99 ± 0.03^a^	0.023	0.790	0.040

Chronic, low-level inflammation in skeletal muscle was investigated by measuring the expression levels of the cytokines *IL-6*, *IL-1β*, and *TNF-α* in gastrocnemius muscle by qRT-PCR analysis (Figures 3E–G). In this study, aging significantly induced inflammation. Interestingly, PQQ decreased the expression of cytokines, particularly *IL-6* and *TNF-α*, suggesting that PQQ could alleviate chronic inflammation caused by aging (inflammaging). We also examined the expression of satellite cell regulatory genes (*Pax7* and *Myf5*) and mitochondrial biogenesis regulatory genes (*PGC-1α* and *Tfam*); however, no improvement effects of PQQ on the expressions of these genes were observed in this study (Supplementary Figure S3).

### 3.6 Serum analysis

The results of serum analyses are presented in Table 4. In this study, compared with the young group, the old group had significantly lower ALB (*p* = 0.021) and, although not significant, a drop in T-CHO levels (*p* = 0.053), indicating that they were undernourished, probably owing to a decline in protein and lipids absorption. PQQ-supplemented aged mice showed no improvement in ALB levels but showed increased T-CHO levels (*p* = 0.7 vs young; *p* = 0.4 vs old), suggesting that PQQ has a tendency to reduce malnutrition by improving fat absorption.

**TABLE 4:** Serum analysis in young, old, and old + PQQ mice. Data indicate the mean ± SEM (n = 6 per group). Values with different superscript letters in a row are significantly different among groups (one-way ANOVA, all pairwise multiple comparisons with the Holm–Sidak method, *p* < 0.05), whereas the values without superscript letters are non-significant to each other. ABL, albumin; T-CHO, total cholesterol; CRE, creatinine; AST, aspartate aminotransferase; ALT, alanine aminotransferase; CK, creatine kinase; Na, natrium; Ca, calcium; Mg, magnesium; TG, triglyceride.

	Young	Old	Old + PQQ	*p* Values		
				Young vs old	Young vs old + PQQ	Old vs old + PQQ
ALB (g/dL)	3.1 ± 0.1^a^	2.6 ± 0.2^b^	2.6 ± 0.2^b^	0.021	0.020	1.000
T-CHO (mg/dL)	135 ± 10	111 ± 5	127 ± 18	0.053	0.702	0.417
CRE (mg/dL)	0.11 ± 0.00	0.12 ± 0.01	0.12 ± 0.00	0.470	0.227	0.504
AST (IU/L)	41 ± 2	45 ± 4	44 ± 4	0.367	0.474	0.837
ALT (IU/L)	19 ± 2	16 ± 2	17 ± 2	0.213	0.484	0.669
CK (IU/L)	77 ± 4	80 ± 6	74 ± 10	0.697	0.804	0.641
Na (mEq/L)	149.7 ± 0.6	151.5 ± 0.6	152.0 ± 0.6	0.110	0.117	0.322
Ca (mg/dL)	9.3 ± 0.1	9.5 ± 0.1	9.6 ± 3.3	0.199	0.112	0.114
Mg (mg/dL)	3.0 ± 0.1	3.2 ± 0.1	3.3 ± 0.2	0.656	0.618	0.440

Creatine kinase (CK) leaks into the blood when muscle tissue is damaged (Baird et al., 2012). Higher CK levels in the blood serum may indicate bone- or muscle-related diseases, such as muscular dystrophy, polymyositis, or dermatomyositis (Amato and Dumitru, 2002). The old + PQQ group had the lowest CK levels among the three groups, although the difference was not statistically significant. Creatinine (CRE) level is an indicator of kidney function, whereas aspartate aminotransferase (ASL) and alanine aminotransferase (ALT) are indicators of liver function. Our results revealed no serious kidney or liver dysfunction in aged mice compared with younger mice. The mineral absorption status (Na, Ca, and Mg) in aged mice was also the same as that in normal mice.

### 3.7 Cell proliferation, senescence, and differentiation in D-gal-induced C2C12 cells

**FIGURE 4:**
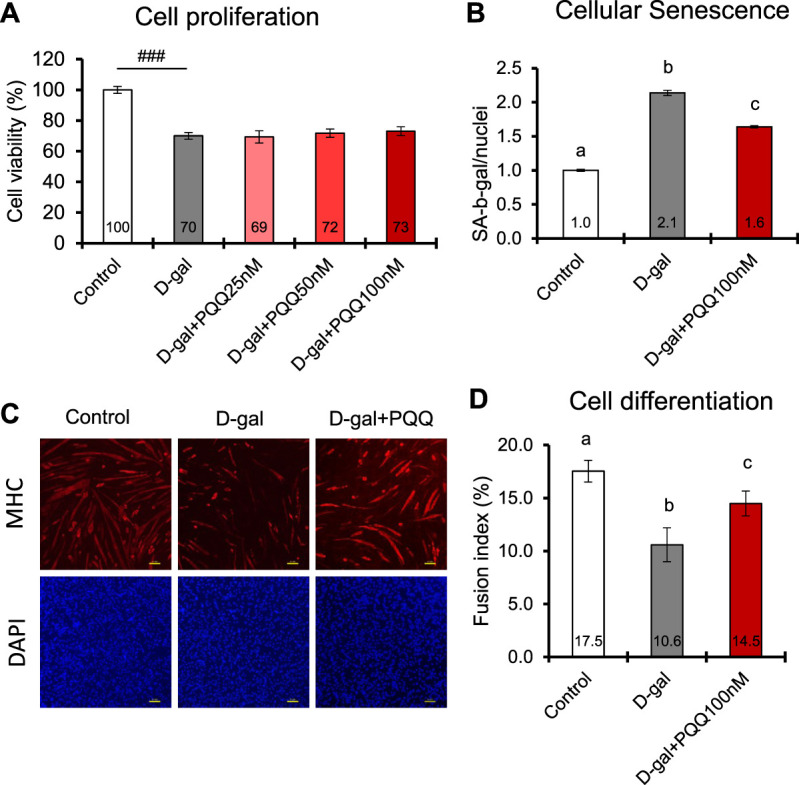
Effects of PQQ on **(A)** cell proliferation **(B)** cellular senescence, and **(C,D)** cell differentiation of aging-induced mouse myoblast cells. **(C)** The representative images of immunostaining of myosin heavy chain (MHC) in differentiated C2C12 myotubes control cells, and D-gal-induced cells treated with or without 100 nM PQQ. Top panel show images of myotubes (red), whereas bottom panel show images of nuclei (blue). **(D)** The fusion index (%) was determined with the number of myosin heavy chains expressing myotubes with greater than 2 nuclei divided by the total number of nuclei. The data indicates mean ± SEM (n = 8). Bars with different letters demonstrate significant differences among treatment conditions (one-way ANOVE, all pairwise multiple comparisons with the Holm-Sidak method, *p* < 0.05). Hashtags denote a statistically significant difference (Paired *t*-test, ###*p* < 0.001) when compared with the control.

D-gal-induced C2C12 cells are known to mimic skeletal muscle atrophy during the aging process (Zhang D. et al., 2020). In this study, we used 20 g/L D-gal to induce senescence and apoptosis in C2C12 myoblast cells (Chen et al., 2020). The results showed that D-gal induction significantly decreased cell proliferation and cell differentiation while increasing the number of senescent cells compared with control healthy cells (Figure 4). Intervention with PQQ in D-gal-induced cells did not affect cell proliferation (Figure 4A) but significantly attenuated cell senescence (Figure 4B) and improved cell differentiation, as indicated by FI (Figures 4C, D). Myoblasts fuse to form multinucleated structures, known as myotubes. As more myoblasts fuse into myotubes, the CSA of the myotubes increases, contributing to muscle fiber thickness. These results are consistent with those of our animal experiments, where PQQ supplementation increased the CSA of aged mouse muscle fibers, leading to better muscle strength and performance.

### 3.8 Cellular ROS and mitochondria change in D-gal-induced C2C12 cells

**FIGURE 5:**
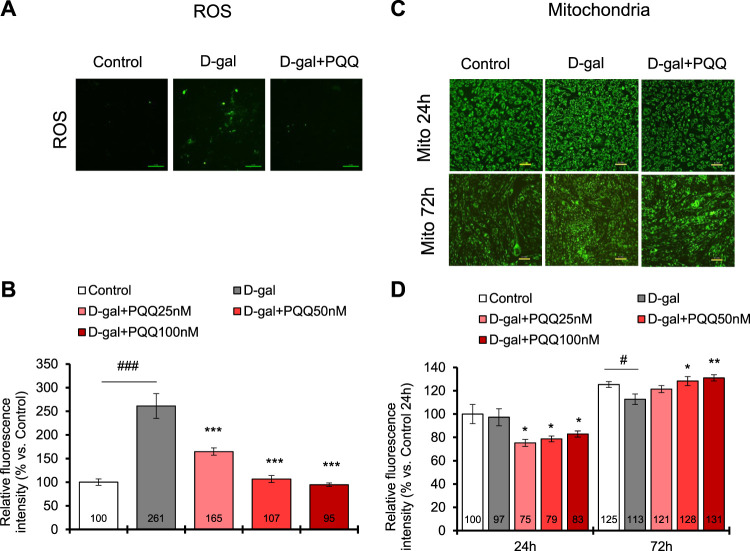
PQQ decreased total ROS levels, and improved mitochondria (Mito) in D-gal-induced C2C12 myoblast cells. **(A)** The representative images of ROS level (green) in control cells, D-gal-induced cells, and D-gal-induced cells supplemented with PQQ after 24 h treatment. **(B)** The quantification of total ROS levels was performed using a microplate reader with a fluorescent filter. The data indicates mean ± SEM (n = 8). **(C)** The representative images of mitochondria (green) in control cells, D-gal-induced cells, and D-gal-induced cells supplemented with PQQ after 24 and 72 h of treatments. **(D)** The quantification of mitochondria intensity was performed using fluorescence imaging analysis. The data indicates mean ± SEM (n = 10). Asterisks denote a statistically significant difference paired *t*-test, (*p < 0.05; **p < 0.01; ***p < 0.001) when compared with only D-gal-treated cells. Hashtags denote a statistically significant difference paired *t*-test, (#*p* < 0.05; ###*p* < 0.001) between D-gal-treated cells and control.

Excess ROS cause cellular damage and contribute to senescence (Davalli et al., 2016). In D-gal induced C2C12 cells, the ROS level was increased by 2.6-fold compared with control healthy cells; however, with PQQ intervention, the ROS levels were significantly reduced (Figures 5A, B). Remarkably, PQQ at 50 and 100 nM concentrations could reverse the ROS levels in D-gal induced C2C12 cells to that of control healthy cells. Additionally, since increased ROS levels contribute to impaired mitochondrial function (Chapman et al., 2019), we investigated the changes in mitochondrial number in D-gal-induced cells with and without PQQ (Figures 5C, D). After 24 h of treatment, we observed a significant reduction in mitochondrial function in D-gal + PQQ treated cells compared with control healthy cells; however, this was not observed in D-gal-treated cells. We continued to observe the cells 72 h after treatment, and surprisingly, the number of mitochondria effectively increased with PQQ treatment, particularly at 50 and 100 nM concentrations, whereas without PQQ treatment, the number of cells significantly decreased (Figure 5D). RT-qPCR analysis revealed that 100 nM PQQ inhibited the reduction of the mRNA expressions of mitochondrial biogenesis regulatory genes *PGC-1α* and *Tfam* in D-gal-induced cells (Supplementary Figure S4). Furthermore, mitophagy was detected using fluorescence staining (Supplementary Figure S5A). The results demonstrated that mitophagy was highly observed in cells induced with D-gal for 24 h, regardless of the presence or absence of PQQ, compared to in control cells. After 72 h, non-treated and D-gal + PQQ-treated cells showed a significant reduction in mitophagy, but this was not seen in D-gal-treated cells (Supplementary Figure S5B). Additionally, mitochondrial function was examined by measuring NAD^+^ levels after 24 and 72 h of treatment. D-gal-induced cells showed significantly reduced levels of total NADH/NAD^+^ and NAD^+^. The presence of PQQ in D-gal-induced cells prevented this reduction (Supplementary Figure S6; Supplementary Table S4). These results suggest that in D-gal induce cells, PQQ enhanced the clearance of dysfunctional mitochondria by mitophagy and promoted mitochondrial biogenesis, which improved mitochondria function in myoblast cells.

### 3.9 Cell proliferation and monolayer permeability in D-gal-induced IEC6 cells

**FIGURE 6:**
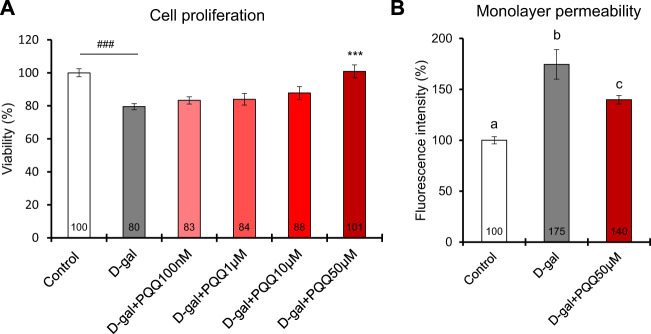
Effects of PQQ on **(A)** cell proliferation and **(B)** monolayer permeability of aging-induced rat small intestine epithelial cells. A total of 48 h of D-gal treatment significantly reduced cell viability (n = 8) and monolayer permeability, expressed as a percentage of FITC-dextran-4 fluorescence intensity (n = 4), compared non-treated control cells. Addition of 50 µM PQQ in D-gal-treated cells significantly improved cell proliferation. The data indicate mean ± SEM. Asterisks denote a statistically significant difference (Paired *t*-test, ***p < 0.001) when compared with D-gal-treated cells. Hashtags denote a statistically significant difference (Paired *t*-test, ###*p* < 0.001) when compared with the control. Bars with different letters indicate significant differences among groups (one-way ANOVA, all pairwise multiple comparisons with the Holm–Sidak method, *p* < 0.05).

IEC6 cells induced with 20 g/L D-gal for 48 h showed a significant reduction in cell viability compared to non-treated control cells (Figure 6A). Interestingly, the addition of PQQ led to an improvement in cell proliferation of D-gal-treated cells in which the 50 µM dose significantly reversed the cell viability compared to non-treated cells. We performed a monolayer permeability assay to test epithelial barrier function influenced by D-gal treatment with and without PQQ intervention. The results demonstrated an increase in FITC-dextran fluorescence intensity in D-gal-treated cells (Figure 6B), indicating barrier dysfunction of IECs. Trans-endothelial electrical resistance of each cell condition was also measured (Supplementary Figure S7); lower resistance in D-gal-treated cells indicated disruption of monolayer health. PQQ intervention reduced barrier dysfunction of the epithelial monolayer.

## 4 Discussion

### 4.1 Aging progression and body conditions in aged mice

In this study, we used naturally aging C57BL/6J male mice because C57BL/6J mice older than 18 months are commonly chosen for sarcopenia studies (Xie et al., 2021). Moreover, the functional capabilities of aging C57BL/6J mice have been characterized to map phenotype of human-equivalent ages (Yanai and Endo, 2021). Two groups of 84-week-old (∼21 months of age) mice were fed a normal chow diet with or without PQQ supplementation for 10 weeks, and 9-week-old (∼2 months of age) mice were used as a young control group (n = 6 animals per group). After 8 weeks of housing, we evaluated the phenotypic characteristics of aging mice. Aged mice progressively developed hair loss, skin dermatitis, skeletal muscle inflammation, muscle atrophy and weakness, body fat loss, and fluid accumulation. They showed no change in body weight but showed a decrease in fat mass and body fat ratio after 2 months of feeding. These findings are consistent with the fact that mice reach their peak body weight (weight gain ceases) at 20–24 months of age, which corresponds to 70–80 years in humans, after which body weight gradually declines; in contrast, fat mass tends to decrease between 17 and 24 months of age in mice (Pappas and Nagy, 2019).

Based on body composition and conditions, we identified that aged mice experienced malnutrition. According to the American Society of Parenteral and Enteral Nutrition and the Academy of Nutrition and Dietetics, the presence of two or more characteristics among weight loss, reduced nutritional intake, muscle wasting, muscle weakness, body fat loss, and fluid accumulation can be diagnosed as malnutrition (Fischer et al., 2015). Furthermore, serum ALB, T-CHO, and lymphocytes are traditional biomarkers for assessing clinical malnutrition in older adults (Ignacio de Ulíbarri et al., 2005; Okamura et al., 2008; Ng et al., 2017). A significant lower ALB and marginally significant lower T-CHO (*p* = 0.053) levels were also evident in aged mice in this study, other than muscle weakness, body fat loss, and body fluid accumulation conditions. Malnutrition is often caused by insufficient consumption of nutrients. Aging processes can affect physiology and metabolism, resulting in changes in the nutritional condition of older individuals (Amarya et al., 2015; Norman et al., 2021). Although aged mice had a significantly higher food intake than the other groups, we speculated that malnutrition could have occurred due to impaired nutrient absorption within the intestinal lumen caused by aging. Body fat accumulation was observed in the young group, which is a natural process that provides energy reserves and insulation around visceral organs. However, the old group exhibited rapid loss of body fat. We hypothesized that due to aging, nutrients were not efficiently absorbed, energy was no longer metabolized from ingested nutrients, and triglycerides from stored body fat were used as energy sources. As a result, the amount of stored body fat decreased rapidly. However, it is also important to note that, besides the changes in nutritional condition, the stress induced by the anesthesia and multiple test procedures at the end of the study could potentially lead to body fat loss.

### 4.2 PQQ alleviated aging progression

In this study, PQQ (20 mg/kg/day) was supplemented through mouse food pellets. Based on body surface area calculation (Nair and Jacob, 2016), the human equivalent dose was determined as 1.6 mg/kg, or 96 mg per 60 kg of an adult. The recommended human dose of PQQ is 20 mg/day, and clinical investigations have reported that PQQ is safe at doses up to 100 mg/day (Turck et al., 2017). We found that a 20 mg/kg PQQ dose in aged mice prevented rapid loss of body fat and muscle atrophy and weakness, mitigated chronic inflammation in skeletal muscle, and improved skin and coating conditions. Additionally, D-gal treatment has been used to accelerate aging in both *in vivo* and *in vitro* models, which induced oxidative stress, inflammatory cytokines, and senescence associated-genes (Azman and Zakaria, 2019); therefore, we also investigated the effects of PQQ in D-gal-induced myoblast cells and IECs. A study in mice fed with radiolabeled [14C]-PQQ revealed that approximately 62% of PQQ was readily absorbed in the lower intestine before 81% was excreted in the urine within 24 h. The remaining PQQ was distributed mostly in the kidney, liver, blood, and other tissues (Smidt et al., 1991). Due to this finding, we designated an appropriate dose of PQQ in myoblast cells, 500–1,000 times lower compared to that in IECs in our *in vitro* studies. Here, we discuss the molecular mechanisms underlying the effects of PQQ in these age-associated conditions.

PQQ is a cofactor that plays an important role in cellular metabolism. It has been shown to promote mitochondrial function and biogenesis (Chowanadisai et al., 2010; Saihara et al., 2017) and enhance lipid metabolism (Qiu et al., 2021; Zhang et al., 2022). Owing to these properties, PQQ reduces body fat accumulation (Devasani et al., 2020; Mohamad Ishak et al., 2021) when there is excess energy intake, thereby promoting human health by attenuating obesity progression (Devasani and Majumdar, 2019; Mohamad Ishak and Ikemoto, 2023). In the present study, PQQ prevented body fat loss and we hypothesized that this was because of impaired energy metabolism caused by aging. This may indicate that PQQ can maintain metabolic homeostasis, which is essential for proper body function and overall health. Further study is required to validate this hypothesis.

Furthermore, we believe that PQQ can ameliorate malnutrition due to poor intestinal absorption of nutrients in the older population. The intestinal epithelium is composed of a continuous monolayer of IECs for absorption. They are tightly linked to ensure a functional and strong barrier between the internal and external environments (Ternet and Kiel, 2021). Aging can contribute to changes in the intestinal barrier, leading to a condition known as “intestinal barrier dysfunction” (Funk et al., 2020). Improving IEC proliferation can enhance intestinal homeostasis as this is necessary for maintaining the health and function of the intestinal epithelium (Ternet and Kiel, 2021). Our *in vitro* study revealed that PQQ promoted the proliferation of IEC6 cells and improved epithelial barrier dysfunction, suggesting that PQQ can improve intestinal function. Additionally, a study in piglets has demonstrated that supplementation with PQQ at a low dose of 1.5 mg/kg could effectively improve the performance and development of small intestinal cells (Yin et al., 2019). Additionally, supplementation with 3 mg/kg PQQ can reduce jejunal mucosal inflammatory damage in piglets challenged with *E. coli* K88 by suppressing the NF-κβ pathway and controlling colonic microbiota imbalance (Huang et al., 2020). Thus, we speculate that PQQ can inhibit body fat loss in older adults by improving metabolic dysfunction and small intestinal performance. However, the effects of PQQ on nutrient absorption in the intestine should be thoroughly examined in the future.

**FIGURE 7:**
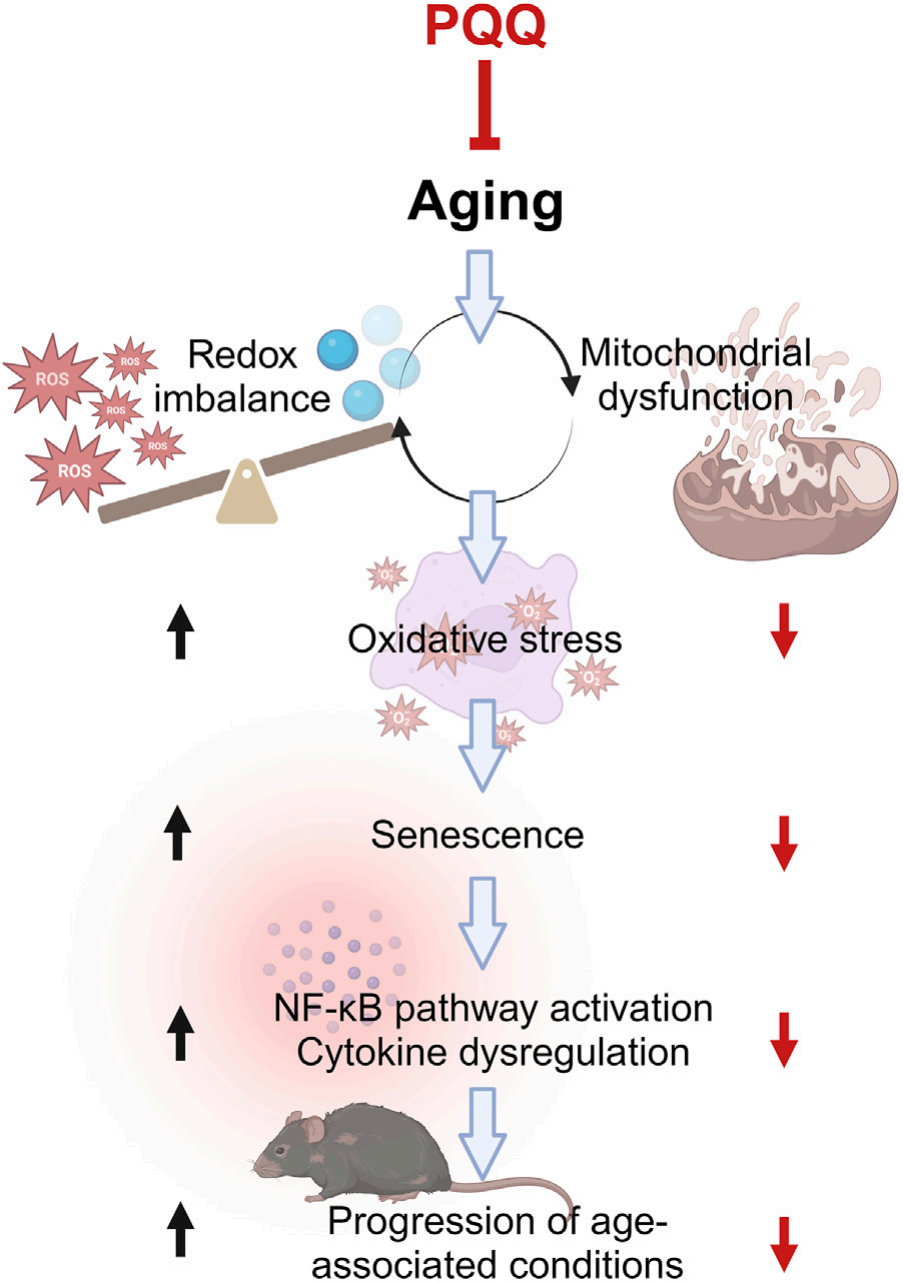
Common molecular pathways associated with aging and low-grade inflammation or “inflammaging.” Aging progresses when ROS is higher than antioxidants, leading to mitochondrial dysfunction and *vice versa*. This condition increases oxidative stress, resulting in cellular senescence and subsequently inflammaging. The inflammaging is caused by cytokine dysregulation, which is activated by the NF-κB pathway, leading to the progression of various age-associated conditions such as frailty and sarcopenia. PQQ attenuates oxidative stress and cellular senescence by improving redox imbalance and mitochondrial dysfunction, which eventually hinders aging progression including muscle weakness and atrophy. The illustration was created with BioRender.com.

Aging has a significant impact on muscle mass, strength, and function and has serious implications for physical performance. Muscle atrophy, defined as a decrease in the size or mass of muscle tissue, occurs when the protein catabolic rate exceeds protein anabolism. This can result from age-related malnutrition (Norman et al., 2021) and inflammaging—the chronic, low-grade inflammation that develops in an advanced age (Antuña et al., 2022). Recently, the common molecular association between aging and low-grade inflammation has become more prominent (Rea et al., 2018). As summarized in Figure 7, a redox imbalance occurs when the number of oxidant molecules, particularly ROS, exceeds the number of antioxidant molecules. Redox imbalance has long been linked with aging. The overproduction of ROS leads to the development of oxidative stress (Harman, 1956). Approximately 90% of intracellular ROS are generated in the mitochondria during energy production, and ROS leakage can cause damaging mutations in mitochondrial genes. Increasing age further contributes to mitochondrial dysfunction and reduced mitophagy activity, i.e., the removal of mitochondria (Diot et al., 2016; Sun et al., 2016). The generated ROS can react with a myriad of macromolecules, such as lipids, proteins, and nucleic acids, which contribute to DNA oxidation and cell membrane damage. These phenomena trigger cellular senescence, which arrests cell proliferation and contributes to the senescence-associated secretory phenotype (SASP) (Campisi, 2013). SASP-secreting cells particularly produce pro-inflammatory cytokines, such as IL-1, IL-6, and TNF-α, downstream from the NF-κβ pathway. This dysregulation of cytokine expression or inflammaging has been associated with age-associated conditions such as muscle weakness and age-related diseases (Rea et al., 2018).

A previous study has shown that PQQ ameliorates skeletal muscle atrophy induced by denervation (Ma et al., 2019). Our study also demonstrated that PQQ attenuated muscle atrophy caused by aging by increasing fiber size without altering fiber number, particularly fast-twitch muscle fibers. This is consistent with the results of our *in vitro* study using D-gal-induced mouse myoblast cells, in which PQQ increased cell differentiation but had no effect on cell proliferation. This resulted in improvements in muscle strength and function observed in the hanging and pole tests, respectively. Moreover, PQQ reduced the high expressions of cytokines such *as IL-6, IL-1β*, and *TNF-α* in the skeletal muscle of aged mice, indicating the alleviation of inflammaging. This was also supported by myoblast cell experiments, in which PQQ reduced cellular ROS, improved mitochondria number and function, and attenuated senescent cell number. We believe that PQQ inhibits NF-κβ signaling that mediates the expression of pro-inflammatory cytokines, as reported by previous studies (Yang et al., 2014; Ma et al., 2019; Huang et al., 2020). Furthermore, in 3-days D-gal treatment with PQQ, the number of mitochondria was significantly reduced after 24 h of treatment before increasing, similar to the mitochondria of healthy cells. We reasoned that PQQ improved mitophagy activity to clear damaged mitochondria at an early stage and enhancing mitochondrial biogenesis and function. Other studies have demonstrated that PQQ promotes autophagy, leading to reduced microglial neuroinflammation (Zhang Q. et al., 2020; Gao et al., 2021) and extended longevity in *Caenorhabditis elegans* (Yang et al., 2021). Therefore, the effects of PQQ on mitophagy activity in aged cells should be studied further.

### 4.3 PQQ as a healthy aging active ingredient

PQQ is a food ingredient that has garnered considerable interest in the field of anti-aging medicine and for overall wellbeing owing to its potential role in promoting mitochondrial function and cellular integrity (Akagawa et al., 2016; Jonscher et al., 2021; Mohamad Ishak and Ikemoto, 2023). Although PQQ is not a miracle anti-aging solution, it has been studied for its potential benefits, some of which may be associated with aging and longevity. Research has shown that PQQ improves cognitive brain function for memory learning ability in aged mice (Yamada et al., 2020) and for composite and verbal memory in older adults (Tamakoshi et al., 2023). Additionally, other studies in mice have demonstrated that the antioxidant effects of PQQ ameliorate age-related hearing loss (Gao et al., 2022), prevent estrogen deficiency-induced osteoporosis (Geng et al., 2019), and protect against skin aging (Li et al., 2022).

Here, we discovered that supplementation with PQQ improved malnutrition in aged mice, inhibiting rapid body fat loss, body fluid accumulation, and muscle atrophy and weakness. As shown in Figure 7, PQQ slowed down the aging process in mice, particularly in skeletal muscle, by improving redox imbalance via mitochondrial biogenesis, which reduced oxidative stress and senescent cells. In turn, the overexpression of pro-inflammatory cytokines was suppressed, which reduced the progression of aging phenotypes, including integument conditions and muscle performances. Taken together, our findings suggest that PQQ has anti-aging potential and can be utilized as a nutritional supplement for healthy aging and longevity. Although research on the effects of PQQ on longevity is still in its early stages, increasing evidence suggests that PQQ may positively impact overall health and longevity.

## Data Availability

The original contributions presented in the study are included in the article/Supplementary Material, further inquiries can be directed to the corresponding author.
