# Supplementary material for: Dietary pyrroloquinoline quinone hinders aging progression in male mice and D-galactose-induced cells
Source: Front Aging. 2024 Feb 29;5:1351860. doi: 10.3389/fragi.2024.1351860 (PMC10938241; doi:10.3389/fragi.2024.1351860)
Supplement: Supplementary file 1 [file DataSheet1.DOCX]

Supplementary Material

# Supplementary Data

## Supplementary Methods and Result

### Mice Fecal Analysis

Fecal samples from each mouse were collected four weeks after the start of supplementation. The fecal samples were vacuum-dried at 60 °C for 1 h. After weighing the dry weight, samples were transferred into a 2.0 mL homogenized tube (TOMY SEIKO, Tokyo, Japan) containing an appropriate amount of silicon beads (TOMY SEIKO) and 400 µL distilled water. Triglyceride (TG) concentration was measured using a LabAssay Triglyceride Kit (FujiFilm Wako, Kanagawa, Japan) and total protein was measured using a *Proteostain*-Protein Quantification Kit (Dojindo, Kumamoto, Japan) for each fecal sample. The results of fecal analysis are shown in Supplementary Table 2. Although the data were not statistically significant, undigested TG (fat) and total protein were higher in the fecal samples of aged mice than in those of younger mice, and PQQ only decreased TG in the feces.

### mRNA Expression Analysis

Myoblasts were seeded at a density of 30,000 cells/well into 12-well plates. After 24 h, D-gal (20 g/L) was added with or without 100 nM PQQ. The cells were incubated for 24 or 72 h at 37 °C under 5% CO_2_. Total RNA was extracted from each sample using RNA Miniprep (Zymo Research, CA, USA), following the manufacturer’s protocol. RNA samples were diluted with DNase/RNase-free water to an appropriate concentration, and 100–140 ng of RNA was used as a template for RT-qPCR using the One-Step TB Green PrimeScript PLUS RT-PCR kit (Takara Bio, Shiga, Japan). The primer sets used in this study are listed in Supplementary Table 3. *PGC-1α* and *Tfam* gene expression was calculated with *B-actin* as a reference gene similarly to as mentioned in the main text methods. The result presented in Supplementary Figure 5 indicates that D-gal treatment significantly reduced the mRNA expressions of mitochondrial biogenesis regulatory genes *PGC-1α* and *Tfam*. However, supplementation with PQQ in D-gal-induced cells inhibited the decrease in the expression of these genes.

### Mitophagy Measurement

To examine mitophagy activity, myoblast cells were stained with 100 nM Mitophagy Dye (Dojindo) and incubated at 37 °C for 30 min. After washing the cells with serum-free medium, they were treated with D-gal, with or without PQQ, in serum-free medium for 24 or 72 h. To observe the co-localization of mitophagy and lysosomes, the medium was discarded, and cells were stained with 1 μL Lyso Dye (Dojindo) at 37 °C for 30 min. The cells were then washed with HBSS buffer without phenol red (FujiFilm Wako). The cells were then observed under a fluorescence microscope. Images of Mitophagy Dye-stained mitochondria and lysosomes were captured using RFP and GFP filters, respectively. Images were analyzed using the ImageJ software to measure the mitophagy density. As shown in Supplementary Figure 5, the results demonstrated D-gal induced mitophagy after 24 h of treatment, regardless of the absence or presence of 100 nM PQQ. After 72 h, mitophagy was significantly reduced in D-gal-treated cells with the addition of PQQ, suggesting that PQQ enhanced the clearance of damaged mitochondria in treated cells.

### Total NADH/NAD^+^ and NAD^+^ Measurements

D-gal (20 g/L) with and without 100 nM PQQ was added to myoblast cells (5 × 10^5^ cells) and NAD+/NADH levels were measured after 24 and 72 h of culture. NAD+/NADH levels were evaluated using the NAD/NADH Assay Kit-WST (Dojindo) in treated cells after removing the culture supernatant. The results showed that treatment with D-gal resulted in a decrease in the total NAD^+^/NADH and NAD^+^, and the presence of PQQ inhibited this decrease.

# Supplementary Figures and Tables

## Supplementary Figures

**(A)**

**
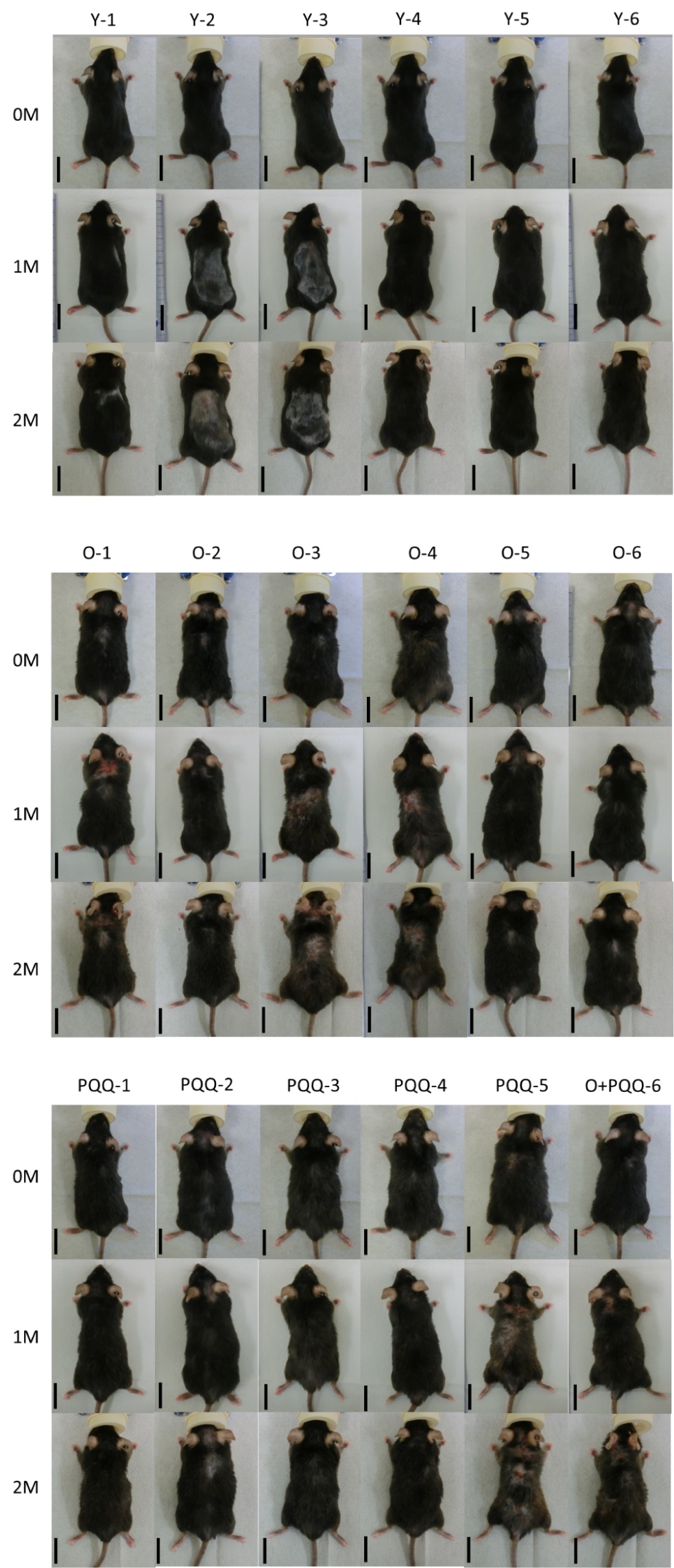
**

**(B)**

**
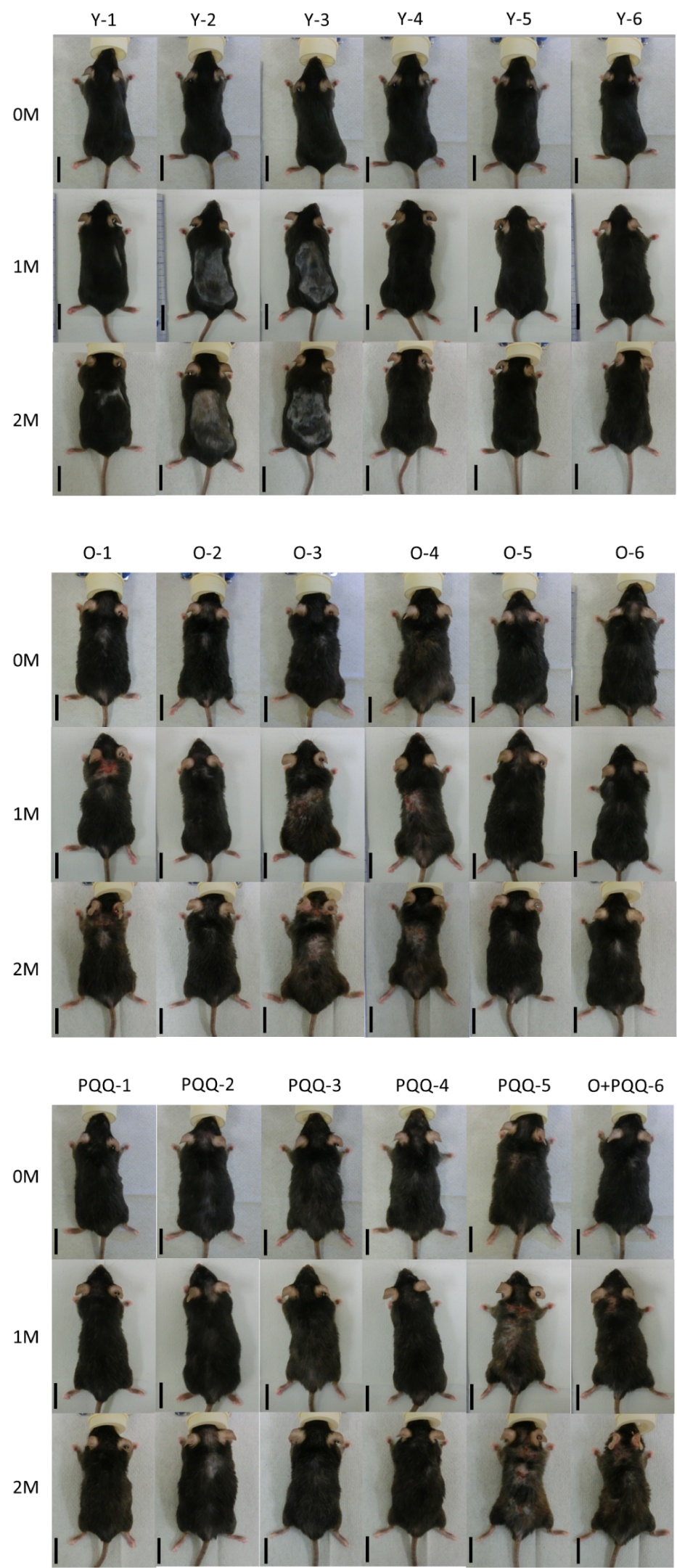
**

**(C)**

**
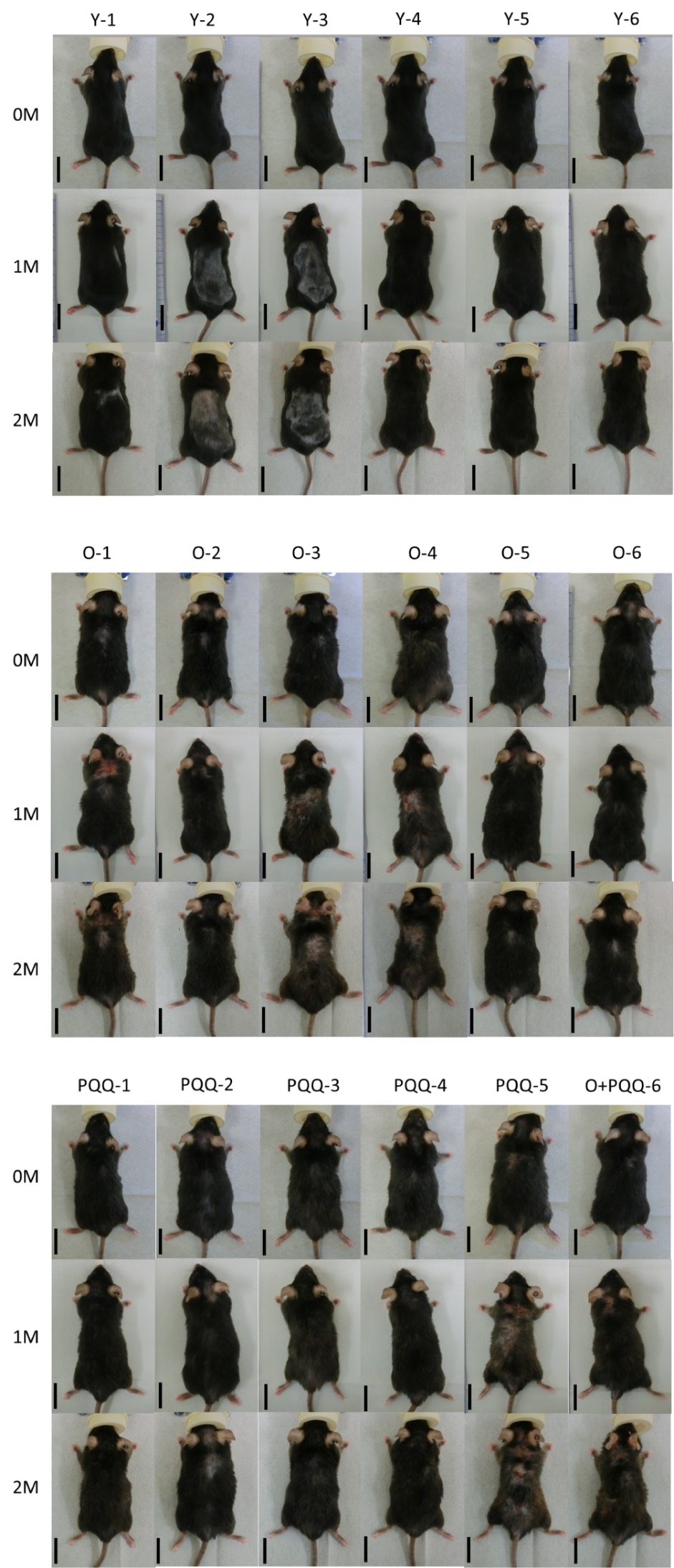
**

**Supplementary Figure 1.** Images of each mouse from the young (A), old (B), and old+PQQ groups (C) at the starting point (0M), 4 weeks (1M), and 9 weeks (2M) after starting dietary supplementation. Scale bars, 2 cm.


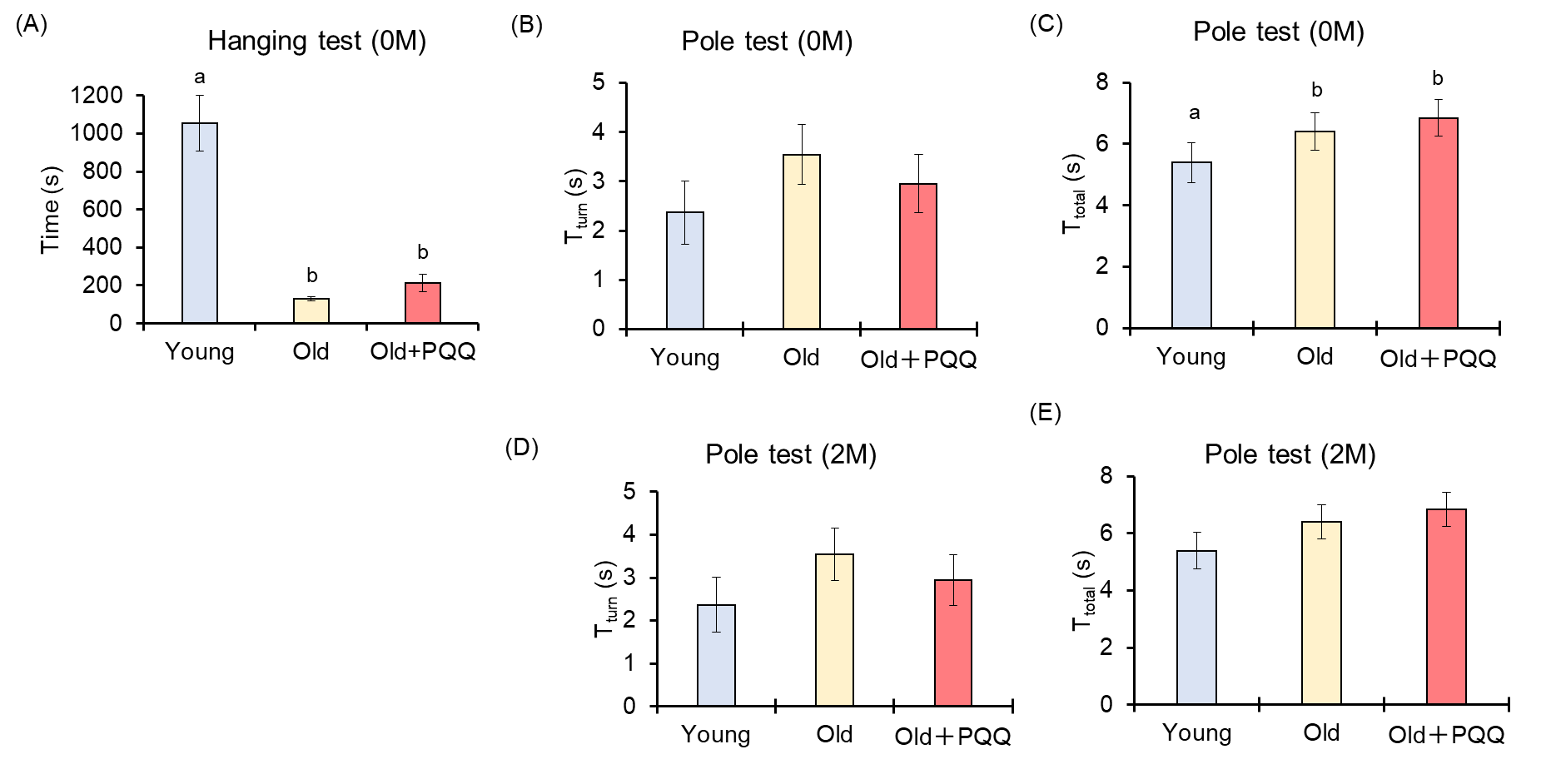


Supplementary Figure 2. (A) The latency time of each group during the wire hanging test performed at the beginning of the study (0M). (B, D) The T_turn_ and (C, E) T_total_ of each group during the pole test at the beginning of the study (0M) and after 2 months of diet consumption (2M), respectively. The data indicates mean ± SEM, (n = 6). Bars with different letters demonstrate significant differences (one-way ANOVA, all pairwise multiple comparisons with the Holm-Sidak method test, P < 0.05).


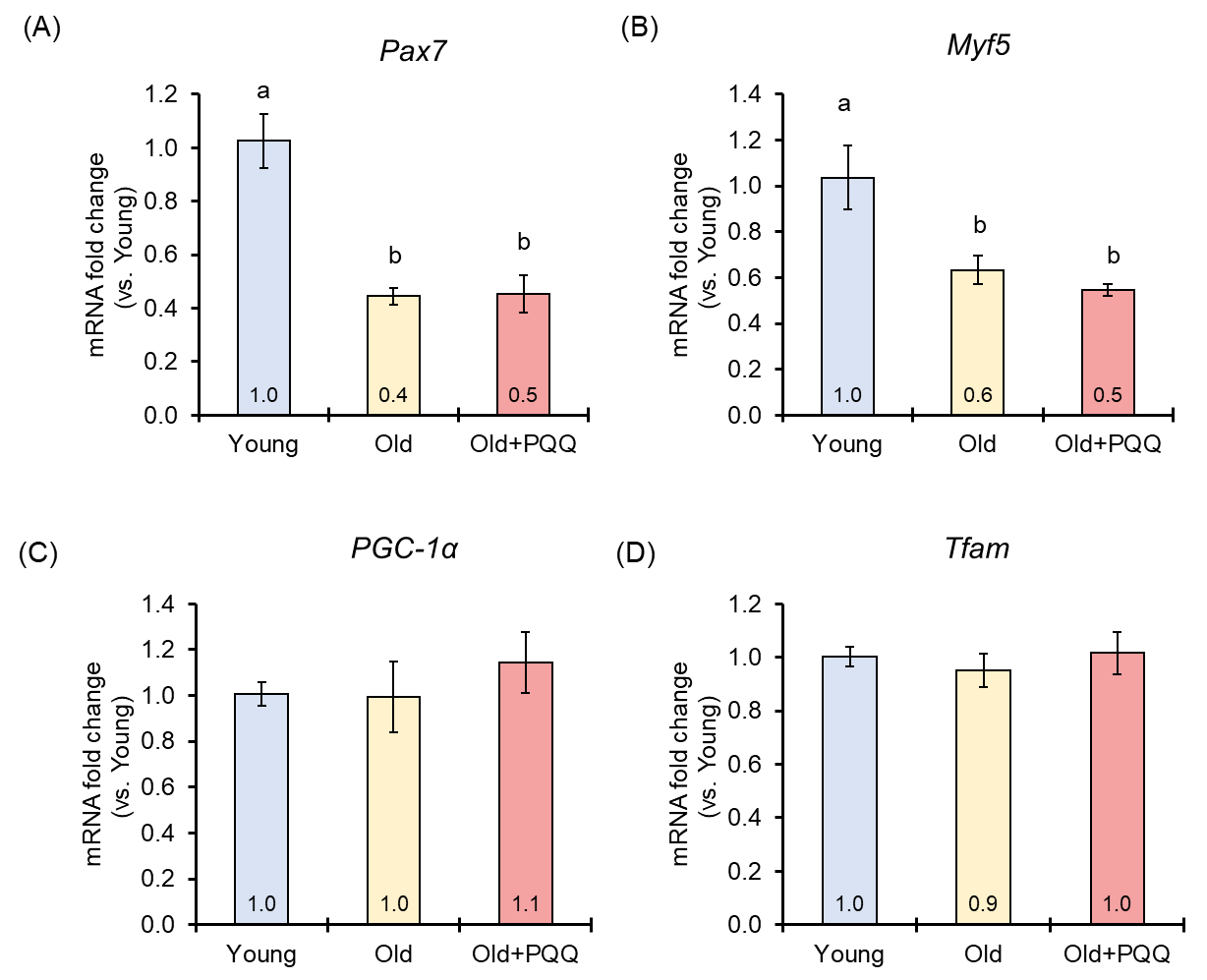


Supplementary Figure 3. Analysis of the mRNA expressions of satellite cell regulatory genes (A) *Pax7* and (B) *Myf5* in mice gastrocnemius mucle. Analysis of the mRNA expressions of mitochondrial biogenesis regulatory genes (C) *PGC-1α* and (D) *Tfam* in mice gastrocnemius muscle. The data indicates mean ± SEM, (n = 6). Bars with different letters demonstrate significant differences (P < 0.05).


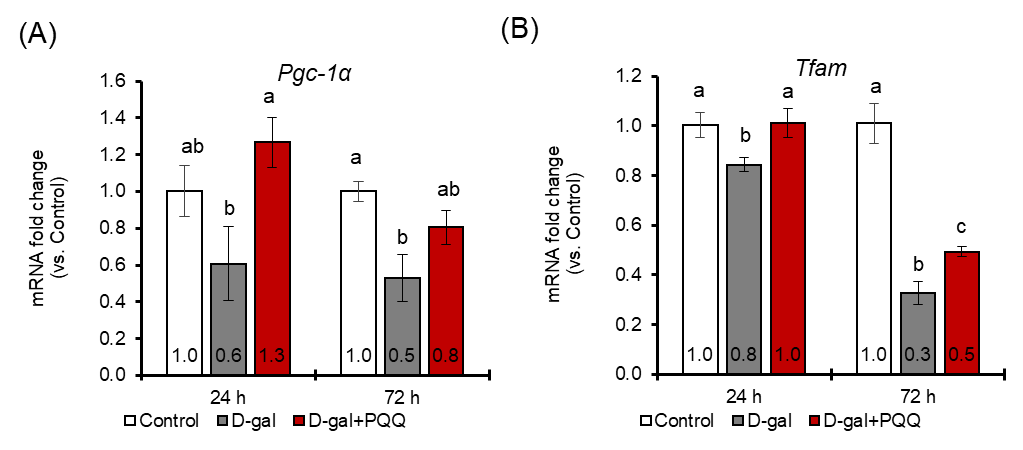


Supplementary Figure 4. Analysis of the mRNA expressions of mitochondrial biogenesis regulatory genes (C) *PGC-1α* and (D) *Tfam* in myoblast cells. The data indicate mean ± SEM, (n = 4). Bars with different letters demonstrate significant differences (P < 0.05).


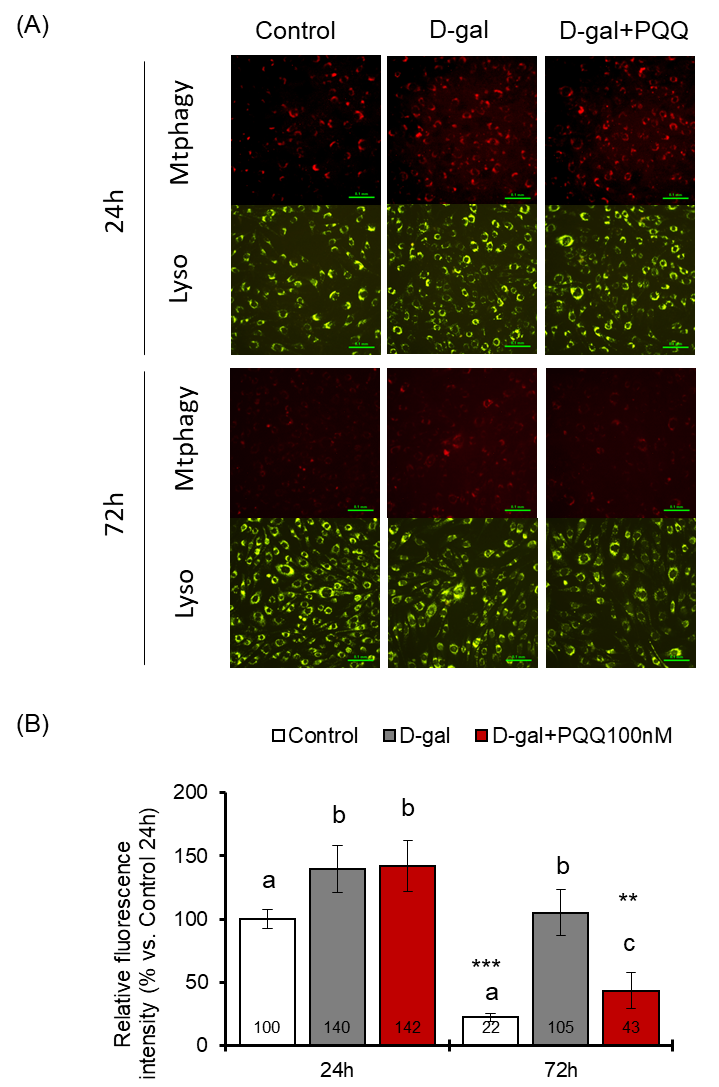


Supplementary Figure 5. (A) Representative images of Mitophagy Dye-stained mitochondria (red) and Lyso Dye-stained lysosome (green) in control, D-gal-induced, and D-gal-induced cells supplemented with PQQ after 24 and 72 h of treatment. (B) The quantification of mitophagy was performed using fluorescence imaging analysis. The data indicate mean ± SEM, (n = 10). Bars with different letters demonstrate significant differences (P < 0.05) among treatment conditions at the same time point. Asterisks denote a statistically significant difference (* P < 0.05; ** P < 0.01; *** P < 0.001) when compared between 24 and 72 h of the same treatment conditions.


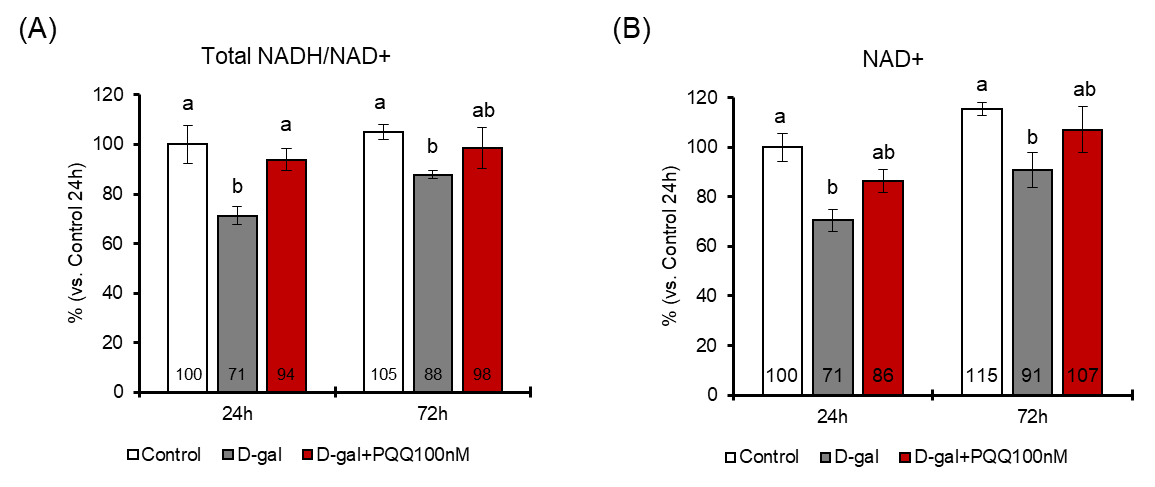


Supplementary Figure 6. The amount of (A) total NAD^+^/NADH and (B) NAD^+^ in myoblast cells after 24 and 72 h of D-gal and D-gal+PQQ treatments. The data indicates mean ± SEM, (n = 4). Bars with different letters demonstrate significant differences (P < 0.05) among treatment conditions at the same time point.


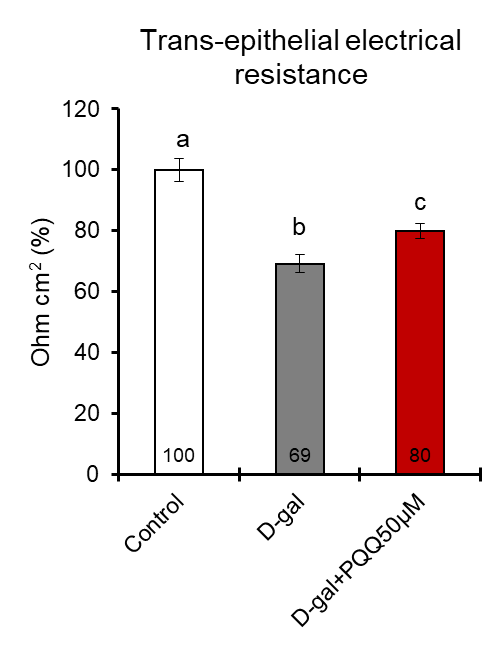


Supplementary Figure 7. Trans-endothelial electrical resistance measured following 48 h of exposure to D-gal showing a significant decrease in resistance. PQQ reduced the decrease in resistance in the D-gal cells. The data indicate mean ± SEM, (n = 4). Bars with different letters demonstrate significant differences (P < 0.05) among treatment conditions.

## Supplementary Tables

Supplementary Table 1. Clinical signs of integument frailty in aging mice evaluated in this study and their corresponding scores.

| **Integument condition** | **Definition** | **Score** | | |
| --- | --- | --- | --- | --- |
|  |  | **0** | **0.5** | **1** |
| Alopecia | Hair loss – patches of missing fur not caused by fighting or barbering | No hair loss observed | 1 or 2 patches | >2 |
| Dermatitis | Skin inflammation – red and inflamed skin lesion | No skin inflammation observed | 0.5 cm^2^ width red area no skin breaking; very small lesion | >0.5 cm^2^ width or bigger red area, no skin; lesion that is larger |
| Coat condition | Fur thickness | No skin can be seen through the fur | Looking at the mouse from the back up towards its head, the skin can be observed adequately but not from other angles | The skin can be observed through the fur easily through most angles |

Supplementary Table 2. Primer sequences used in the RT-qPCR experiment.

| Gene | Forward primer | Reverse primer |
| --- | --- | --- |
| *PGC-1a* | CGCACAACTCAGCAAGTCCTC | CTTGCTGGCCTCCAAAGTCTC |
| *Tfam* | AAGACCTCGTTCAGCATATAACATT | TTTTCCAAGCCTCATTTACAAGC |
| *Pax7* | GGCACAGAGGACCAAGCTC | GCACGCCGGTTACTGAAC |
| *Myf5* | CTGCTCTGAGCCCACCAG | GACAGGGCTGTTACATTCAGG |
| *Il-6* | TGCAAGAGACTTCCATCCAG | AGTGGTATAGACAGGTCTGTTGG |
| *Il-1β* | GAAATGCCACCTTTTGACAGTG | TGGATGCTCTCATCAGGACAG |
| *TNF-α* | CAGGCGGTGCCTATGTCTC | CGATCACCCCGAAGTTCAGTAG |
| *B-actin* | CTGGCTCCTAGCACCATGAAGAT | GGTGGACAGTGAGGCCAGGAT |

Supplementary Table 3. Fecal analysis of young, old, and old+PQQ mice. Data indicate the mean ± SEM, (n = 6 per group).

|  | **young** | **old** | **old+PQQ** |
| --- | --- | --- | --- |
| TG (mg/dL) | 274 ± 48 | 297 ± 38 | 209 ± 28 |
| Total protein (g/L) | 4.2 ± 1.3 | 5.8 ± 1.3 | 6.1 ± 1.5 |

Supplementary Table 2. Total NAD^+^/NADH and NAD^+^ (nmol/mL) 24 and 72 h after treatment. Data are presented as the mean ± SEM (n=4).

|  | **control** | **D-gal** | **D-gal + PQQ** | **P-value control vs. D-gal** | **P-value control vs. D-gal+PQQ** | **P-value D-gal vs. D-gal+PQQ** |
| --- | --- | --- | --- | --- | --- | --- |
| Total NADH/NAD+ (24 h), nmol/mL | 1.77 ± 0.09 | 1.26 ± 0.07 | 1.66 ± 0.08 | 0.012 | 0.431 | 0.018 |
| Total NADH/NAD+ (72 h), nmol/mL | 1.86 ± 0.04 | 1.55 ± 0.03 | 1.74 ± 0.15 | 0.003 | 0.481 | 0.276 |
| NAD+ (24 h), nmol/mL | 1.60 ± 0.09 | 1.12 ± 0.07 | 1.38 ± 0.07 | 0.016 | 0.137 | 0.069 |
| NAD+ (72 h), nmol/mL | 1.84 ± 0.04 | 1.45 ± 0.11 | 1.71 ± 0.15 | 0.032 | 0.437 | 0.240 |
